# Toxicological Effects of Nickel Chloride on IgA^+^ B Cells and sIgA, IgA, IgG, IgM in the Intestinal Mucosal Immunity in Broilers

**DOI:** 10.3390/ijerph110808175

**Published:** 2014-08-11

**Authors:** Bangyuan Wu, Hengmin Cui, Xi Peng, Jing Fang, Zhicai Zuo, Junliang Deng, Jianying Huang

**Affiliations:** Key Laboratory of Animal Diseases and Environmental Hazards of Sichuan Province, College of Veterinary Medicine, Sichuan Agricultural University, Ya’an 625014, China; E-Mails: wubangyuan2008@163.com (B.W.); pengxi197313@163.com (X.P.); fangjing4109@163.com (J.F.); zzcjl@126.com (Z.Z.); dengjl213@126.com (J.D.); hjy19860316@163.com (J.H.)

**Keywords:** nickel chloride, IgA^+^ B cell, immunoglobulins, small intestine, cecal tonsil, broiler

## Abstract

The objective of this study was to investigate the toxicological effects of dietary NiCl_2_ on IgA^+^ B cells and the immunoglobulins including sIgA, IgA, IgG and IgM in the small intestine and cecal tonsil of broilers by the methods of immunohistochemistry and enzyme-linked immunosorbent assay (ELISA). Two hundred and forty one-day-old avian broilers were randomly divided into four groups and fed on a control diet and three experimental diets supplemented with 300, 600, and 900 mg/kg NiCl_2_ for 42 days. Compared with the control group, the IgA^+^ B cell number and the sIgA, IgA, IgG, and IgM contents in the NiCl_2_-treated groups were significantly decreased (*p* < 0.05 or *p* < 0.01). It was concluded that dietary NiCl_2_ in the excess of 300 mg/kg had negative effects on the IgA^+ ^ B cell number and the abovementioned immunoglobulin contents in the small intestine and the cecal tonsil. NiCl_2_-reduced sIgA, IgA, IgG and IgM contents is due to decrease in the population and/or the activation of B cell. The results suggest that NiCl_2_ at high levels has intestinal mucosal humoral immunotoxicity in animals.

## 1. Introduction

Nickel (Ni) is a ubiquitous trace metal that occurs in soil, water, air, and the biosphere [1]. Cement dusts containing the chemical substance (as Ni or Ni compounds) can spread over a large area via wind, rain and so on, and can accumulate in plants, animals or soil, which can affect the environment and even human health [2,3]. As an important environmental pollutant, Ni can be absorbed by human beings and animals via the respiratory tract, gastrointestinal tract or skin [4]. Uptake of too much Ni can generate toxicity symptoms. It has been proved that higher dosages of Ni are toxic and carcinogenic to humans or animals [5,6]. Acute exposure may also induce neurological symptoms, adrenal insufficiency, hyperglycemia, hepatic toxicity, nephrotoxicity, nasal and lung cancer [7]. It is also reported that dietary Ni has negative effects on laying hens [8] and 3-wk-old male chicks [9]. In addition, Ni and Ni compounds have toxicological effects on the immune system [10]. T lymphocytes, B lymphocytes, natural killer (NK) cells and macrophages are all susceptible to Ni toxicity [11]. However, toxicological effect mechanism of Ni or Ni compounds on intestinal immunity remains to be elucidated at present.

The gastrointestinal tract is one of the main locums where metals (including Ni) are absorbed. The tract is exposed to much higher concentrations of metals due to the daily consumption of food and water. Some studies have shown that Ni intake causes immunotoxicity [5]. Dietary nickel chloride (NiCl_2_) induces intestinal oxidative damage [12] and cecal tonsil apoptosis [13] in broilers. Moreover, the small intestine (duodenum, jejunum, and ileum) is an important component of the mucosal immune system and performs important and unique immune functions. Intestinal epithelia participate in host defense through the interaction with critical components of the mucosal immune system. The cecal tonsil of bird is the largest lymphoid organ of the avian gut-associated lymphoid tissue, which is located in the proximal end of the rectum-cecum-ileum as a part of the intestine [14] and performs important and unique immune functions [15]. Besides, the cecal tonsil, as a secondary lymphoid organ, plays a sentinel role in immunity by producing antibodies. Diffuse lymphoid tissue and unorganized lymphoid follicles are also appeared in the mucosa and submucosa of the cecal tonsil [16].

Based on the abovementioned references and the fact that studies on the toxicological effects of Ni or Ni compounds on the immunoglobulins in the intestinal mucosal immunity in animals and human being have never been reported to date, the aims of the present study were to investigate the humoral immune function of the intestinal mucosal immunity by detecting the distribution and populations of IgA^+^ B cells in the small intestine (duodenum, jejunum and ileum) and the cecal tonsil by immunohistochemistry staining, and the changes in secretory IgA (sIgA), immunoglobulin A (IgA), immunoglobulin G (IgG) and immunoglobulin M (IgM) contents in the small intestinal mucosa and the cecal tonsil by enzyme-linked immunosorbent assay (ELISA) kits.

## 2. Materials and Methods

### 2.1. Chickens and Diets

Two hundred and forty one-day-old healthy avian broilers were randomly divided into four groups with 60 broilers in each group. Broilers were housed in cages with electrically heated units and provided with water as well as undermentioned diets *ad libitum* for 42 days.

A corn–soybean basal diet formulated by the National Research Council (1994) [17] was the control diet. NiCl_2_·6H_2_O (Chengdu Kelong Chemical Reagent Company, Chengdu, China) was mixed into the corn–soybean basal diet to produce experimental diets with 300 mg/kg, 600 mg/kg and 900 mg/kg of NiCl_2_, respectively.

### 2.2. Immunohistochemical Examination for IgA^+^ B cells in the Small Intestine (Duodenum, Jejunum and Ileum) and the Cecal Tonsil

Five chickens in each group were humanely sacrificed for gross examination at 14, 28 and 42 days of age. Duodenum, jejunum, ileum and cecal tonsil were collected and fixed in 10% neutral buffered formalin, and then processed and trimmed, embedded in paraffin.

IgA^+^ B cells were localized in the duodenum, jejunum, ileum and cecal tonsil by immunohistochemistry. The immunohistochemical staining and counting were performed as described by Liu *et al.* [15]. Slices were dewaxed in xylene, rehydrated through a graded series of ethanol washes, washed in distilled water and phosphate buffer saline (PBS) and then blocked for endogenous peroxidase by incubation with 3% H_2_O_2_ in methanol for 15 min. The sections were subjected to antigen retrieval procedure by microwaving in 0.01 M sodium citrate buffer pH 6.0. Additional washing in PBS was performed before the next 30 min of incubation at 37 °C in 10% normal goat serum. The slices were incubated overnight at 4 °C with the diluted (1:100) primary antibodies. The antibodies used were polyclonal mouse anti-chicken IgA heavy chains (8330-01, SouthernBiotech, Birmingham, Alabama, USA). For negative controls, the slices received PBS in place of the primary antibody. After washed in PBS, the slices were exposed to 1% biotinylated secondary antibody goat anti-mouse IgG (ZB-0314, ZSGB-BIO, Beijing, China) for 1 h at 37 °C, and then incubated with the HRP-streptavidin (ZB-2305, ZSGB-BIO, Beijing, China) for 30 min at 37 °C. To visualize the immunoreaction, sections were immersed in diaminobenzidine hydrochloride (DAB). The slices were monitored microscopically and stopped by immersion in distilled water, as soon as a brown color staining was visualized. Slices were lightly counterstained with hematoxylin, dehydrated in ethanol, cleared in xylene and mounted.

IgA^+^ B cells were counted by a computer-supported imaging system connected to a light microscope (AX70, Olympus Optical Co., Ltd, Tokyo, Japan) with an objective magnification of × 40. Then IgA^+^ B cells were quantified by Image-Pro Plus 5.1 (Media Cybernetics, Rockville, MD, USA) image analysis software. For each tissue, five random fields of the five slices at the same place of the intestinal region or cecal tonsil were quantified (corresponding approximately to five fields at 40 × magnification). Results were expressed as the average of positive cells per area. The IgA^+^ B cells positive cells in the crypt and in the middle regions of villi were counted separately.

### 2.3. Determination of the sIgA, IgA, IgG and IgM Contents in the Small Intestine and Cecal Tonsil by ELISA

The mucosal supernatant of the duodenum, jejunum, ileum and the cecal tonsil were prepared and detected as described by Wu *et al.* [12] and Liu *et al.* [15]. The supernatant was immediately assayed for the sIgA, IgA, IgG and IgM contents in the small intestinal mucosa and the cecal tonsil by enzyme-linked immunosorbent assay (ELISA). Immunoglobulin contents were quantified using the sIgA (DZE40206), IgA (DZE40073), IgG (DZE40070) and IgM (DZE40069) ELISA kits specific for chicks. The sIgA, IgA, IgG, and IgM contents were determined by the standard curve and expressed as μg per mL.

### 2.4. Statistical Analysis

Data of the control group and three NiCl_2_-treated groups were statistically evaluated with SPSS/16.0 software package programme for Windows. Hypothesis testing methods included one-way analysis of variance (ANOVA) followed by least significant difference test. *p* < 0.05 was considered as statistical significance. All results were expressed as means ± standard error (*x* ± SE), representing five broilers in each group.

### 2.5. Ethic Statement

The animal protocols used in this work and all procedures of the experiment were performed in compliance with the laws and guidelines of Sichuan Agricultural University Animal Care and Use Committee (Approval No: 09ZA072).

## 3. Results

### 3.1. Changes in the IgA^+^ B Cells in the Small Intestine (Duodenum, Jejunum, Ileum) and Cecal Tonsil

**Figure 1:**
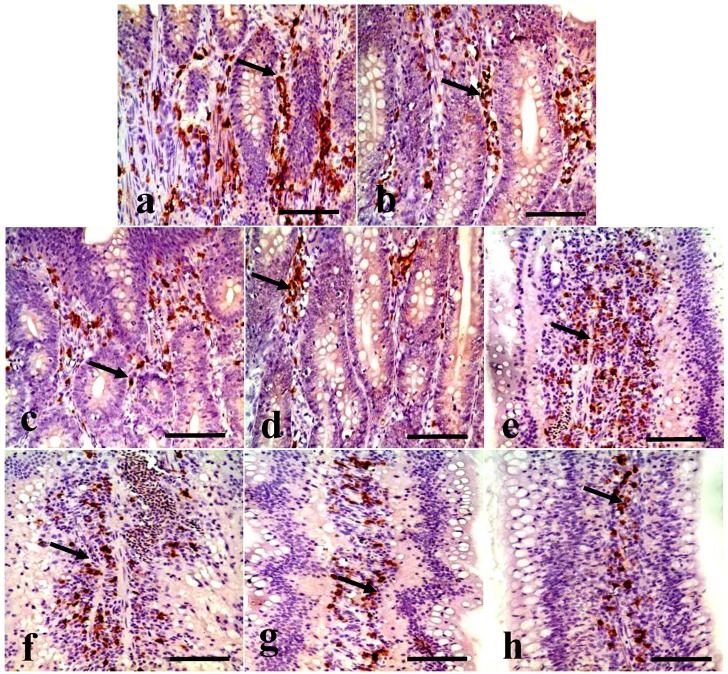
IgA^+^ B cells in the crypts and lamina propria of villi in the duodenum at 42 days of age. (**a**) The duodenal crypts in the control group. (**b**) The duodenal crypts in the 300 mg/kg group. (**c**) The duodenal crypts in the 600 mg/kg group. (**d**) The duodenal crypts in the 900 mg/kg group. (**e**) The duodenal lamina propria in the control group. (**f**) The duodenal lamina propria in the 300 mg/kg group. (**g**) The duodenal lamina propria in the 600 mg/kg group. (**h**) The duodenal lamina propria in the 900 mg/kg group. (SABC, bar = 50 μm).

**Figure 2:**
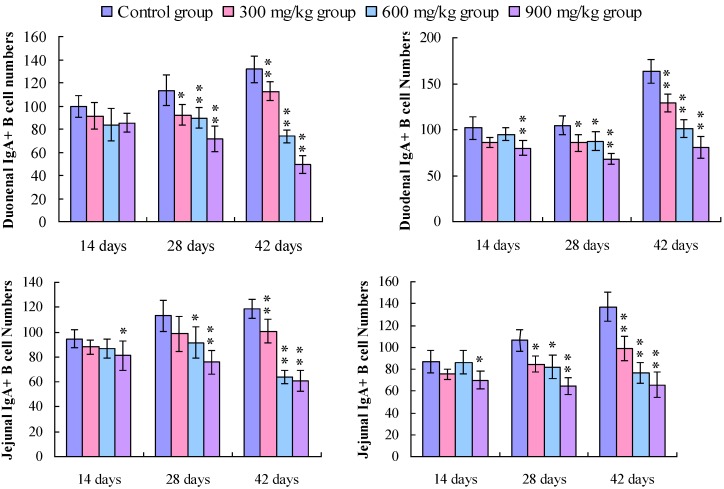
Change in the IgA^+^ B cell number in the lamina propria (LP) of the duodenum and jejunum.

Changes in the IgA^+^ B cells in the duodenum IgA^+^ B cells were mainly distributed in the crypts and the lamina propria of villi in the duodenum. The positive cells were stained brown (arrow, Figure 1). The number of IgA^+^ B cells was significantly decreased in the duodenal crypts and the lamina propria of the 300, 600, and 900 mg/kg groups (Figure 1). In Figure 2, the number of IgA^+^ B cells in the crypts and lamina propria was significantly decreased (*p* < 0.05 or *p* < 0.01) in the 300, 600, and 900 mg/kg groups at 28 and 42 days of age, and in the 900 mg/kg group at 14 days of age.

**Figure 3:**
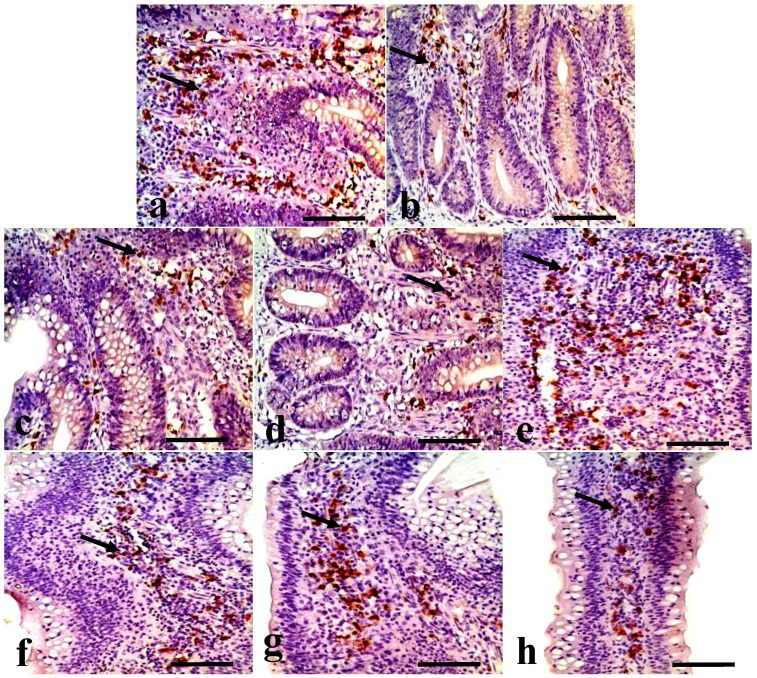
IgA^+^ B cells in the crypts and lamina propria of villi in the jejunum at 42 days of age. (**a**) The jejunal crypts in the control group. (**b**) The jejunal crypts in the 300 mg/kg group. (**c**) The jejunal crypts in the 600 mg/kg group. (**d**) The jejunal crypts in the 900 mg/kg group. (**e**) The jejunal lamina propria in the control group. (**f**) The jejunal lamina propria in the 300 mg/kg group. (**g**) The jejunal lamina propria in the 600 mg/kg group. (**h**) The jejunal lamina propria in the 900 mg/kg group. (SABC, bar = 50 μm).

Changes in the IgA^+^ B cells in the jejunum IgA^+^ B cells mainly distributed in the crypts and the lamina propria of villi in the jejunum. The positive cells were stained brown (arrow, Figure 3). In Figure 3, the IgA^+^ B cells number was significantly decreased in the jejunal crypts and the lamina propria of the 300, 600, and 900 mg/kg groups. The counting of the IgA^+^ B cells in the crypts and lamina propria were also significantly decreased (*p* < 0.05 or *p* < 0.01) in the 300 mg/kg group at 42 days of age, in the 600 mg/kg at 28 and 42 days of age, and in the 900 mg/kg group from 14 to 42 days of age (Figure 2).

Data are presented with the means ± standard error (n = 5 × 5); *p < 0.05, **p < 0.01, compared with the control group; Data were analyzed by variance (ANOVA) test of SPSS 16.0 software.

**Figure 4:**
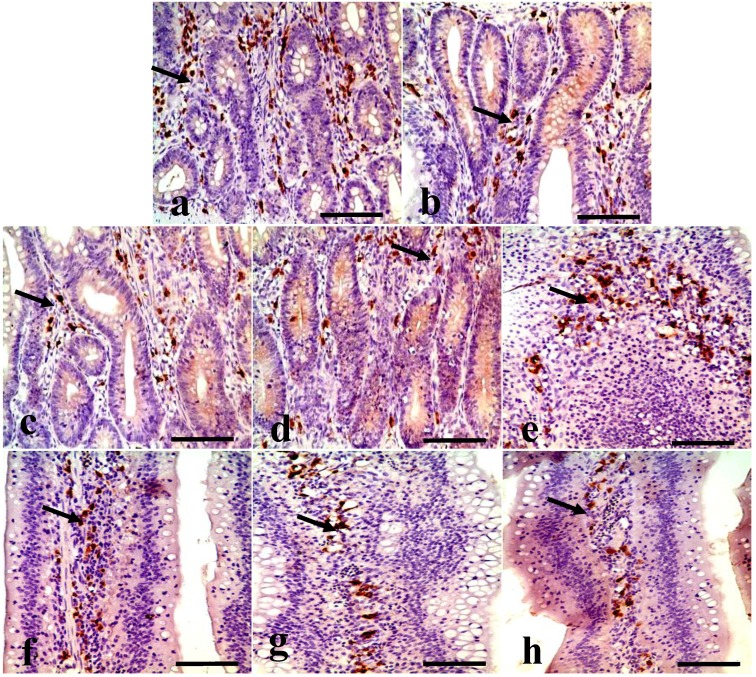
IgA^+^ B cells in the crypts and lamina propria of villi in the ileum at 42 days of age. (**a**) The ileac crypts in the control group. (**b**) The ileac crypts in the 300 mg/kg group. (**c**) The ileac crypts in the 600 mg/kg group. (**d**) The ileac crypts in the 900 mg/kg group. (**e**) The ileac lamina propria in the control group. (**f**) The ileac lamina propria in the 300 mg/kg group. (**g**) The ileac lamina propria in the 600 mg/kg group. (**h**) The ileac lamina propria in the 900 mg/kg group. (SABC, bar = 50 μm).

**Figure 5:**
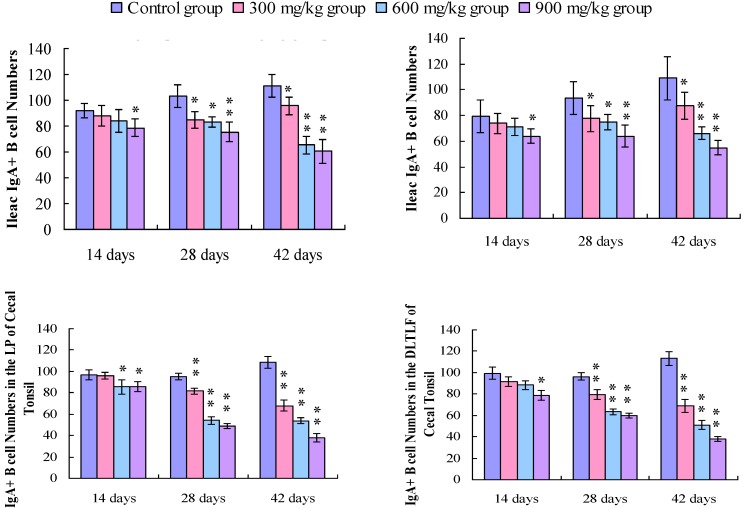
Change in the IgA^+^ B cell number in the lamina propria (LP) of the ileum and the LP, the diffuse lymphoid tissues and lymphoid follicles (DLTLF) of the cecal tonsil.

Changes in the IgA^+^ B cells in the ileum IgA^+^ B cells mainly distributed in the crypts and the lamina propria of villi in the ileum. Figure 4 showed that the positive cells were stained brown (arrow). The IgA^+^ B cell number was significantly decreased in the ileac crypts and the lamina propria of the 300, 600, and 900 mg/kg groups (Figure 4). Also, the counting of the IgA^+^ B cells in the crypts and lamina propria was significantly decreased (*p* < 0.05 or *p* < 0.01) in the 900 mg/kg group at 14 days of age, and in the 300 mg/kg, 600 mg/kg, and 900 mg/kg groups at 28 and 42 days of age when compared with that of the control group (Figure 5).

Data are presented with the means ± standard error (n = 5 × 5); *p < 0.05, **p < 0.01, compared with the control group; Data were analyzed by variance (ANOVA) test of SPSS 16.0 software.

**Figure 6:**
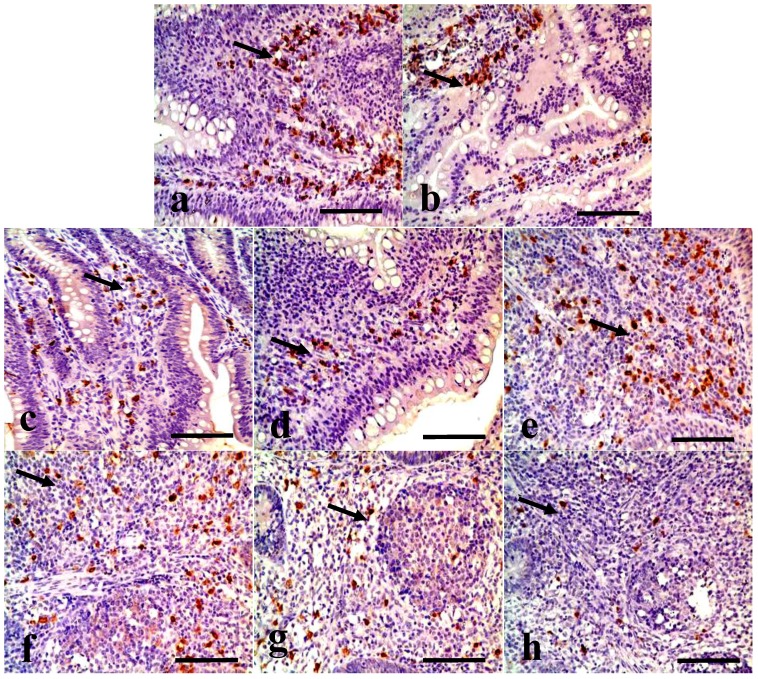
IgA^+^ B cells in the lamina propria and the diffuse lymphoid tissues and lymphoid follicles of the cecal tonsil at 42 days of age. (**a**) The cecal tonsil in the control group. (**b**) The cecal tonsil in the 300 mg/kg group. (**c**) The cecal tonsil in the 600 mg/kg group. (**d**) The cecal tonsil in the 900 mg/kg group. (**e**) The cecal tonsil in the control group. (**f**) The cecal tonsil in the 300 mg/kg group. (**g**) The cecal tonsil in the 600 mg/kg group. (**h**) The cecal tonsil in the 900 mg/kg group. (SABC, bar = 50 μm).

Changes in the IgA^+^ B cells in the cecal tonsil. The IgA^+^ B positive cells were stained brown (arrow), and mainly distributed in the lamina propria, diffuse lymphoid tissues and lymphoid follicles (Figure 6)*.* The number of IgA^+^ B cells was significantly decreased in the lamina propria, diffuse lymphoid tissues and lymphoid follicles (Figure 5). The counting of the IgA^+^ B cells was significantly lower (*p* < 0.05 or *p* < 0.01) in the 300 mg/kg, 600 mg/kg, and 900 mg/kg groups than that in the control group from 14 to 42 days of age, except 300 mg/kg group in the LP, and 300 and 600 mg/kg groups in the DLTLF at 14 days of age, as shown in Figure 5.

### 3.2. Changes in the sIgA Contents in the Small Intestine and Cecal Tonsil

**Figure 7:**
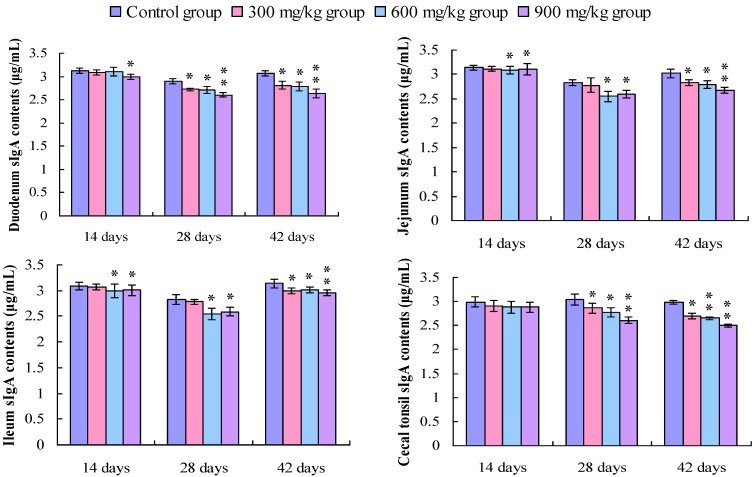
Change in the sIgA contents (μg/mL) in the intestinal mucosa and cecal tonsil in broilers.

The sIgA contents in the duodenum and the cecal tonsil were significantly lower (*p* < 0.05 or *p* < 0.01) in the 300, 600, and 900 mg/kg groups than those in the control group from 14 to 42 days of age, except the cecal tonsil at 14 days of age. The sIgA contents in the jejunum and the ileum were decreased (*p* < 0.05 or *p* < 0.01) in the 600 and 900 mg/kg groups at 14 days of age, and in the 300, 600, and 900 mg/kg groups at 28 and 42 days of age (Figure 7).

Data are presented with the means ± standard error (n = 5); *p < 0.05, **p < 0.01, compared with the control group; Data were analyzed by variance (ANOVA) test of SPSS 16.0 software.

### 3.3. Changes in the IgA Contents in the Small Intestine and the Cecal Tonsil

**Figure 8:**
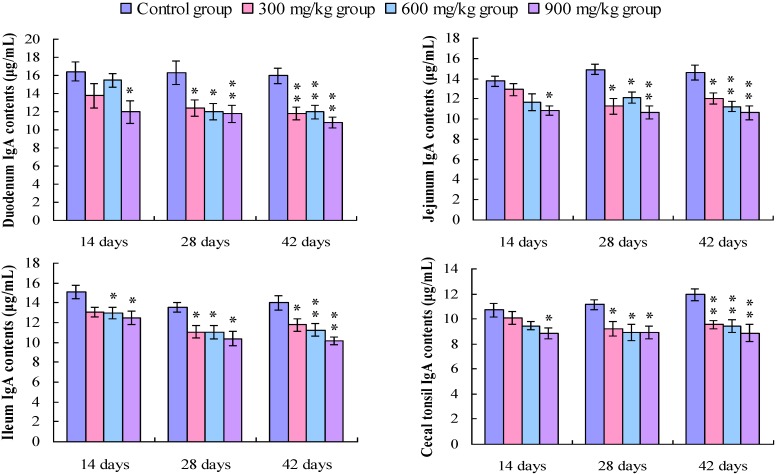
Change in the IgA contents (μg/mL) in the small intestine and cecal tonsil in broilers.

Figure 8 shows that the IgA contents in the duodenum, jejunum and cecal tonsil were significantly decreased (*p* < 0.05 or *p* < 0.01) in the 900 mg/kg group at 14 days of age, and in the 300, 600, and 900 mg/kg groups at 28 and 42 days of age in comparison with those of the control group. The IgA contents in the ileum were significantly decreased (*p* < 0.05 or *p* < 0.01) in the 600 and 900 mg/kg groups at 14 days of age, and in the 300, 600, and 900 mg/kg groups at 28 and 42 days of age.

Data are presented with the means ± standard error (*n* = 5); *p < 0.05, **p < 0.01, compared with the control group; Data were analyzed by variance (ANOVA) test of SPSS 16.0 software.

### 3.4. Changes in the IgG Contents in the Small Intestine and Cecal Tonsil

**Figure 9:**
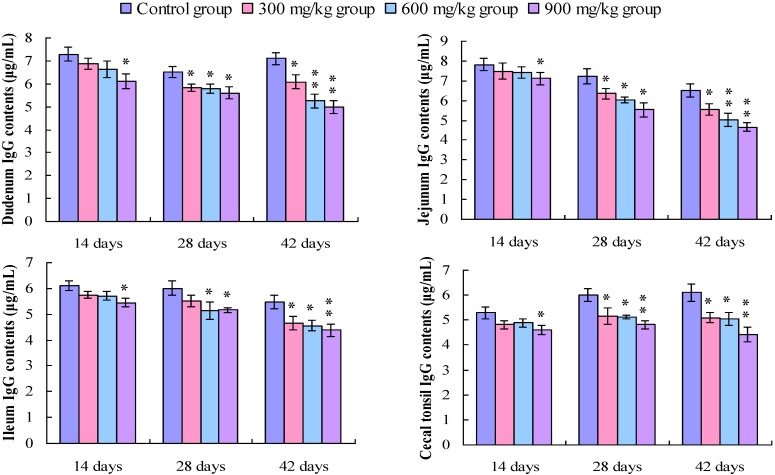
Change in the IgG contents (μg/mL) in the small intestine and cecal tonsil in broilers.

The IgG contents in the duodenum and the cecal tonsil were significantly lower (*p* < 0.05 or *p* < 0.01) in the 900 mg/kg group at 14 days of age, and in the 300, 600, and 900 mg/kg groups at 28 and 42 days of age than those in the control group. The IgG contents in the jejunum were significantly decreased (*p* < 0.05 or *p* < 0.01) in the 300, 600 and 900 mg/kg groups at 28 and 42 days of age. In the ileum, the IgG contents were significantly lower (*p* < 0.05 or *p* < 0.01) in the 900 mg/kg group at 14 days of age, and in the 600 and 900 mg/kg groups at 28 days of age, and in the 300, 600 and 900 mg/kg groups at 42 days of age, as shown in Figure 9.

Data are presented with the means ± standard error (*n* = 5); *p < 0.05, **p < 0.01, compared with the control group; Data were analyzed by variance (ANOVA) test of SPSS 16.0 software.

### 3.5 Changes in the IgM Contents in the Small Intestine and Cecal Tonsil

**Figure 10:**
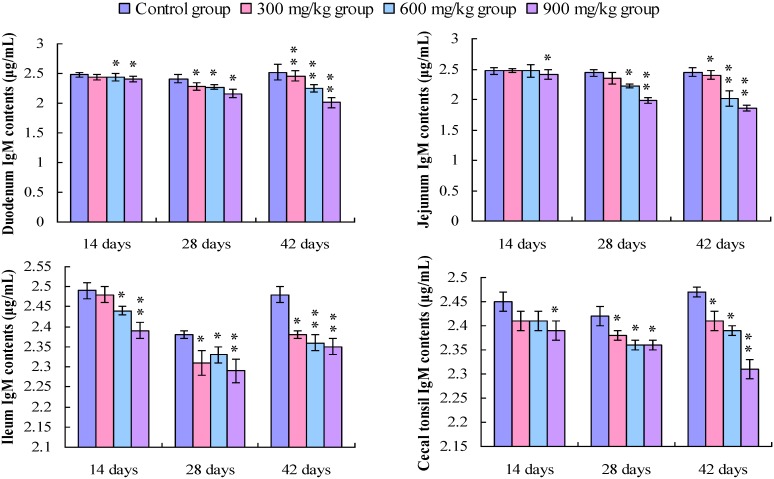
Change in the IgM contents (μg/mL) in the small intestine and cecal tonsil in broilers.

As shown in Figure 10, the IgM contents in the duodenum, jejunum and cecal tonsil were lower (*p* < 0.05) in the 900 mg/kg group at 14 days of age, and were significantly lower (*p* < 0.05 or *p* < 0.01) in the 300, 600, and 900 mg/kg groups at 28 and 42 days of age than those in the control group, except the IgM in the jejunum in the 300 mg/kg group at 28 days of age. The IgM contents in the ileum were significantly decreased (*p* < 0.05 or *p* < 0.01) in the 600 and 900 mg/kg groups at 14 days of age, and in the 300, 600 and 900 mg/kg groups at 28 and 42 days of age.

Data are presented with the means ± standard error (*n* = 5); *p < 0.05, **p < 0.01, compared with the control group; Data were analyzed by variance (ANOVA) test of SPSS 16.0 software.

## 4. Discussion

It is well known that small intestine (duodenum, jejunum, and ileum) and cecal tonsil play an important role in the mucosal immunity system. The mucosal immunity system provides antigen-specific protection through B cells/antibodies and T cells. B cells and antibodies compose humoral immunity or antibody-mediated immunity, and T cells compose cell-mediated immunity [18]. The combination of B cells and T cells protects the host against infection. Thus, a success in an adaptive immune response depends on the functions of humoral and cellular immunity as well as the appropriate interactions between them [19]. The subsequent fate of antigen-antibody complexes depends largely on the Fc regions of immunoglobulins (Ig), which determines the biological properties of the different isotypes [20]. Furthermore, immunoglobulin (or antibody) molecules are multifunctional components of the immune system which mediate interactions between antigen molecules and a variety of cellular and humoral effectors [21]. Consequently, the determination of the T or B cell number, the quantitative or qualitative measure of the cytokine and immunoglobulin levels can be used to evaluate the condition of the immune system [22].

The antibodies are mainly produced in mucosal lymphoid tissues, particularly in intestines [23]. B cells in the lamina propria mature into immunoglobulin-producing plasma cells [24]. Some metals have effects on immunoglobulin synthesis [25], and Ni at high concentration is toxic to animals [26]. Ni or Ni compounds exposure can affect the serum immunoglobulins in human [27]. High levels of Ni may depress the circulating antibody response of rats immunized with a viral antigen [28], and reduce the number of cytoplasmic immunoglobulin positive (cIg^+^) cells of the Ni-sensitive patients’ cells in culture [29]. Also, NiCl_2_ can adversely impact primary antibody production in the spleen of mice [30] and NiSO_4_ cause significantly reduce specific antibody-producing splenocytes in mice [31].

However, the abovementioned studies don’t focus on the effects of Ni or Ni compounds on humoral immunity of the mucosal immune system. In the present study, we investigated the toxicological effects of dietary NiCl_2_ on the IgA^+^ B cells and immunoglobulins in the small intestine (duodenum, jejunum and ileum) and cecal tonsil of broilers. The results provided new experimental evidences for understanding the toxic effect mechanism of NiCl_2_ on the intestinal mucosal immunity.

Chicken immunoglobulin gene rearrangement and the expression of surface membrane immunoglobulin of B cell occur during a certain period of embryonic development [32,33,34]. There are three major immunoglobulin classes in the chicken: IgG (IgY), IgM and IgA [35]. IgA is found to be presented in the overwhelming majority of the intestinal plasma cells, which has a typical feature similar to the mammalian IgA. IgA releases secretory IgA (sIgA) into the gut lumen by the transepithelial transport [36]. IgA class switching enables antibody secretion onto the mucosal surfaces [37].

IgA is critical for protecting mucosal surfaces against toxins, viruses and bacteria by means of neutralizing or preventing them binding to the mucosal surface [38], which is similar to mammalian IgA [39]. IgA is synthesized by local plasma cells [40]. Intracellular IgA such as polymeric nature of secretory IgA (sIgA) is particularly important in preventing bacterial or viral infection and pathogenesis [38]. sIgA can also act as a potentiator of the immune response in intestinal tissue by uptaking antigen to dendritic cells [38]. Moreover, sIgA plays a role in the maintenance of mucosal homeostasis, which may also influence the development of systemic immunity and determine the composition of the intestinal microbiota [41]. In general, IgA class switching requires the stimulation of B cells by CD4+ T cells. CD40 ligand (CD40L) and cytokines including interleukin (IL)-4, IL-10, and transforming growth factor (TGF)-β participate in completing of above procession [42]. CD4+ helper T cells act to provide help for production of IgA B-cell while it maintain tolerance to commensal bacteria and possibly other antigens [43]. Class-switched B cells differentiate into IgA-secreting plasmacytoid B cells, which migrates to the intestinal lamina propria (LP) under the influence of IEC-derived chemokines [44]. Also, T helper 2 (Th2) cytokines (IL-4 and IL-6) can result in activation of B lymphocytes and up-regulation of antibody production [45]. However, we have found that NiCl_2_ can reduce the contents of Th2 cytokines including IL-4 and IL-6 in the intestine and cecal tonsil of broilers [46]. In the present study, the s IgA^+^ B cell population, the sIgA and IgA contents in the small intestine and the cecal tonsil were significantly decreased in the NiCl_2_-treated groups. The results indicated that high levels of NiCl_2_ had suppressive effect on the production of IgA^+^ B cells and IgA (sIgA) in the small intestine and cecal tonsil. After binding to the polymeric immunoglobulin receptor (pIgR), polymeric IgA (sIgA) secreted by plasma cells (IgA B cell) translocates to the surface of epithelial cells, and is released with a portion of the pIgR (secretory component, SC) at the apical surface as secretory IgA (sIgA) complexes [47]. Furthermore, there is a close relationship between IgA^+^ B cells and the humoral immune response, which implies that the reduction of IgA^+^ B cell numbers, sIgA and IgA production will finally impact the humoral immune function in the mucosal immunity of the small intestine and the cecal tonsil in broilers. In addition, the increase in the IgA^+^ B cells in the control group from 14 to 42 days of age may relate to the development of small intestines and cecal tonsil.

IgM is the first immunoglobulin expression on B cells surface. IgM plays roles by opsonizing (coating) antigen for destruction and by fixing complement. IgM antibodies are associated with a primary immune response and frequently used to diagnose acute exposure to an immunogen or pathogen [38]. Chicken IgM is structurally and functionally homologous to mammalian counterpart [48], and is the first antibody generation during a primary antibody response. IgM is also the major class of immunoglobulin expression on the surface of chicken B lymphocytes [49]. In the present study, the contents of IgM in the small intestinal mucosa and cecal tonsil were decreased in the 300 mg/kg, 600 mg/kg and 900 mg/kg groups from 14 to 42 days of age, which indicates that dietary NiCl_2_ can impair primary immune response.

IgG, secreted by B cells, is the main antibody isotype in blood [50]. There are four IgG subclasses (IgG1, IgG2, IgG3, and IgG4). IgG antibodies directly contribute to an immune response including neutralization of toxins and viruses [36]. Functionally, IgG is mainly generated in secondary antibody responses and behaves like the mammalian IgG [51]. The present study shows that IgG contents were decreased in the small intestinal mucosa and cecal tonsil in the 600 and 900 mg/kg groups, implying that dietary NiCl_2_ can impact the clearance of pathogens and immune response. A similar study shows that lead can significantly reduce IgG antibody synthesis [52].

## 5. Conclusions

It is concluded that dietary NiCl_2_ in excess of 300 mg/kg reduces the population of IgA^+^ B cells and the contents of sIgA, IgA, IgG and IgM in the small intestine and the cecal tonsil, implying that the humoral immune function in the intestinal mucosal immunity has been impaired in broilers. NiCl_2_-reduced sIgA, IgA, IgG and IgM contents is due to decrease in the population and the activation of B cell. The results suggest that NiCl_2_ at high levels has intestinal mucosal humoral immunotoxicity in animals.
